# Why Do Protein Folding Rates Correlate with Metrics of Native Topology?

**DOI:** 10.1371/journal.pone.0035599

**Published:** 2012-04-27

**Authors:** Patrícia F. N. Faísca, Rui D. M. Travasso, Andrea Parisi, Antonio Rey

**Affiliations:** 1 Centro de Física da Matéria Condensada, Universidade de Lisboa, Lisboa, Portugal; 2 Departamento de Física, Universidade de Lisboa, Lisboa, Portugal; 3 Centro de Física Computacional and Departamento de Física, Universidade de Coimbra, Coimbra, Portugal; 4 Center of Ophthalmology and Vision Sciences, Institute for Biomedical Research in Light and Image (IBILI), Faculty of Medicine, University of Coimbra, Coimbra, Portugal; 5 Departamento de Química Física I, Facultad de Ciencias Químicas, Universidad Complutense, Madrid, Spain; Universitat Autònoma de Barcelona, Spain

## Abstract

For almost 15 years, the experimental correlation between protein folding rates and the contact order parameter has been under scrutiny. Here, we use a simple simulation model combined with a native-centric interaction potential to investigate the physical roots of this empirical observation. We simulate a large set of circular permutants, thus eliminating dependencies of the folding rate on other protein properties (e.g. stability). We show that the rate-contact order correlation is a consequence of the fact that, in high contact order structures, the contact order of the transition state ensemble closely mirrors the contact order of the native state. This happens because, in these structures, the native topology is represented in the transition state through the formation of a network of tertiary interactions that are distinctively long-ranged.

## Introduction

The notion that form and function are intimately related is an old one in biology. In his seminal work “On Growth and Form”, D’Arcy Thomson explored the relation between natural geometries, the dynamics of growth and physical processes in living systems. The motto ‘function follows form’ is a basic principle of biology operating at any hierarchical level of living matter. In particular, at the microscopic level of macromolecules, it specifically means that the function of a protein is determined by its three-dimensional native structure, which is acquired through the process of protein folding.

In the late 1990’s, Plaxco and co-workers made the serendipitous observation that a parameter named contact order (CO), measuring the average sequence separation between all pairs of residues within physical contact in the native structure, is highly correlated with the logarithmic folding rates of small, single domain proteins that fold in a two-state manner [1]. This led to the idea that the native structure is not only a determinant of the biological function for the molecule, but it is also largely responsible for the protein folding rates. Thus a natural question arises which is that of understanding why the CO is able to predict protein folding rates. Actually, the empirical result is somehow puzzling. If one considers that the protein folding process follows the transition state (TS) theory, then the folding rate should be related to the free energy barrier separating the denatured ensemble from the transition state ensemble (TSE) at a given folding temperature, and therefore it should not be directly linked to the native structure. Thus, the influence of the native structure on the folding rate should be related to the fact that the conformations belonging to the TSE must be somehow similar to the native conformation, at least at the level of similarity captured by the CO. In other words, assuming that the CO can be interpreted as a gross metric of the native topology, then the observed correlation suggests that the rate-limiting step in folding is the acquisition of the native topology by the TSE (or at least that a close approximation to the native topology must be realized in the TSE [2]).

This idea prompted the proposal of the “topomer sampling model” (TSM) [3], [4], a theoretical construct that ignores the energetic stabilization of the TSE [3], [4]. Indeed, according to the TSM the dominant contributor to the folding barrier is a diffusive (non-biased) search for a conformational state with the gross overall topology of the native structure. A critical assessment of the TSM by Chan and Wallin showed that an unbiased search for the native topomer amounts to a Levinthal-like process, which is not compatible with the biological timescale of protein folding [5]. In other words, the rate-limiting step in protein folding has an energetic component that cannot be neglected. On the other hand, an analysis based on simple lattice models reported that the slope of the rate-CO dependence is sensitive to the particular spatial orientation of the protein backbone [6].

The analysis by Fersht of the extended nucleation-condensation mechanism [7]–[9] accommodates the observed dependence of folding rates both on the stability and on the topology of the TSE, by showing that tertiary interactions and interactions within elements of secondary structure are equally important in the folding nucleus [7] because of the entropy loss associated with the former. Along a complementary line of research focused on exploring the microscopic origins of protein energetics underlying folding cooperativity, Chan and co-workers [10] showed that the rate-CO dependence was enhanced by the inclusion of effective multi-body interactions.

Despite these and other conceptually attractive theoretical proposals [11], [12], an in-depth analysis of the rate-CO dependence is still missing. This stems in part from difficulties in isolating the effect of CO in folding kinetics experiments. Fulfilling this condition is critical to ensure a correct assessment of rate-CO correlations, because it is known that the folding rate is influenced by other protein properties (e.g. number of native contacts [13], stability [8], [9], [14], [15] and, to a lesser extent, chain length [15], [16]).

Computer simulation of simple protein models, where all these effects can be minimized or actually fully avoided, can contribute to elucidate this problem. Here, we succeed in accomplishing this goal by comparing the folding rates of an extensive number of simple lattice proteins with fixed chain length that are related to one another by circular permutation. A circular permutant (CP) is an engineered protein that results from linking the C- and N-terminus of the polypeptide chain after disrupting the protein backbone at some selected peptide bond. All the CPs resulting from a given ‘parent’ structure form a family whose members have virtually the same overall native structure, but display different backbone connectivity and, as a consequence, different CO values. Furthermore, and despite its simplicity, the adopted lattice framework (i.e. a three-dimensional cubic lattice, where the distance between neighboring residues along the sequence is set equal to the lattice spacing) ensures that for a fixed chain length all the circular permutants will have exactly the same number of native contacts, a condition that is not as easily guaranteed in more detailed, off-lattice protein representations. In addition, simple lattice models have a long tradition in the study of the fundamental aspects of protein stability and folding [17]–[26], allowing statistically accurate computations of thermodynamic and kinetic quantities. Even though the model is a crude representation of the protein topology, it captures the very basic polymeric traits (chain connectivity, excluded volume, etc.) that should underline the CO-rate correlation at a very fundamental level (after all, the CO is essentially a property of the protein backbone).

**Figure 1:**
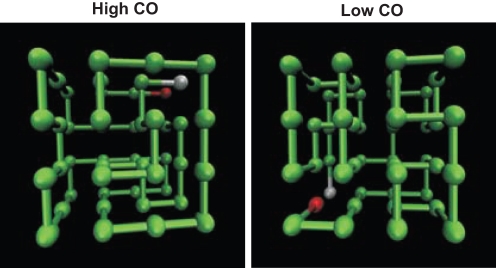
Three-dimensional representation of the lattice proteins that were used to generate the two families of circular permutants with chain length 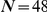. Amino acids are represented by beads of uniform size that occupy the lattice vertices; the peptide bond, which covalently connects amino acids along the polypeptide chain, is represented by sticks with uniform (unit) length corresponding to the lattice spacing. The terminal beads are highlighted.

To be sure that the results we obtain are not dependent on the choice of a particular ‘parent’ structure we investigate two different families of lattice CPs, which are generated (as outlined above) from the lattice proteins represented in Figure 1. These ‘parent’ structures have been obtained through Monte Carlo simulations of homopolymer collapse [27]. By selecting different maximally compact conformations displaying their termini in neighboring lattice positions, one can create as many families of CPs as needed. For any family, the number of CPs is equal to the number of beads (or chain length *N*), which is 48 in this work.

One of the studied families, which we term family H, is generated from the ‘parent’ high-CO native structure shown in the left part of the figure. The other, generated from the low-CO native structure at the right side, is termed family L. The native structures in the two families cover different ranges of the CO parameter. In family H (L) the CO range is 14.1<CO<21.7 (11.7<CO<18.2). It is important to mention that since all our structures have the same chain length *N* it is not relevant to use the *absolute* CO or the *relative* CO (obtained from the former dividing it by *N*). This is yet another advantage of the lattice model employed here.

## Methods

In this work, we take advantage of the simplicity of the model to study the folding process of a very large number of different native structures (a total of 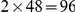 structures). Moreover, we will use the same model to obtain accurate thermodynamic and kinetic information about the folding process; such a goal is not straightforward to accomplish when using more sophisticated (and therefore more realistic) protein models.

To model protein energetics we use a native-centric, or G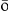-type potential. Accordingly, the energy of a conformation is given by
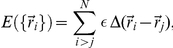
where 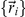 represents the set of bead coordinates defining a given conformation, 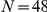 is the chain length, 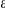 is the (uniform) interaction energy parameter, and 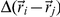 is unity only if beads *i* and *j* form a non-bonded native contact and is zero otherwise. As seen in Figure 1, a non-bonded native contact appears when two non contiguous beads along the protein backbone occupy neighboring lattice positions in the native structure. For the particular lattice model and chain length used in this work, all the native structures are maximally compact 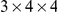 cuboids, as those shown in Figure 1, and have exactly the same number of non-bonded native contacts (which is equal to 57). By using a native-centric model we exclude from the folding process any (putative) effects associated with chemical composition (i.e. protein sequence), and focus our analysis solely on the effects of native structure on the folding process.

**Figure 2:**
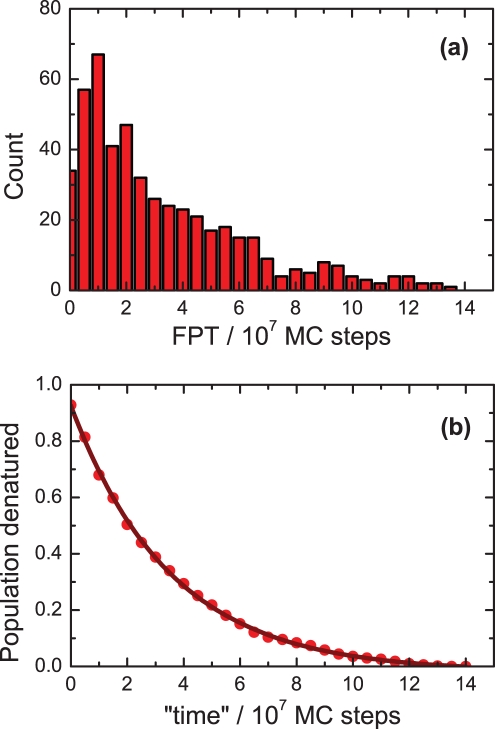
(a) Distribution of the first passage times of folding (FPT) for the 500 independent trajectories of a given CP. (b) "Time" evolution of the denatured state population, obtained by integration of the histogram in the upper graph. The solid line corresponds to the fitting to a single exponential decay.

To sample conformational space we have used a standard Metropolis Monte Carlo (MC) algorithm combined with a local move set that includes corner-flips and end-moves (i.e. displacements of one single bead) and the crankshaft move (which involves the displacement of two beads at the same time). Details of the adopted algorithm can be found elsewhere [28]. For every considered native structure (or CP) we use two different simulation strategies. Firstly, a parallel tempering algorithm is used to obtain a representative equilibrium sampling of protein conformations as a function of temperature; the method simulates several replicas (about 50 in every case) at different temperatures, covering the whole spectrum of protein conformations (ranging from the native state up to the thermally denatured state). On the other hand, to obtain kinetic properties for every CP, we have carried out single (or fixed) temperature MC simulations. To get statistically significant kinetic measurements, we have computed 500 independent folding trajectories; each simulation starts from a different random conformation and stops when the native structure is reached. The individual “folding times” of these folding events, or “first passage times” in more general terms, allow to study the evolution of the population of conformations representative of the unfolded state as a function of MC time (i.e. number of MC steps). One example of this distribution can be observed in Figure 2(a). The histogram can be partially integrated to get the population of unfolded conformations which remains at the considered simulation temperature up to the selected “time”. This is shown in Figure 2(b). We have checked that for all the considered CPs our data can be fitted to a single exponential decay (similar to the solid line shown in Figure 2(b)), which is consistent with two-state folding behavior. The folding rate constant is given by the slope of the linear fitting. The folding rates (in logarithmic scale) are reported in the Results section. The statistical error resulting from the fitting procedure is always smaller than the size of the symbols used in the graphs and therefore is not shown.

The equilibrium simulations resulting from the parallel tempering procedure provide the energy fluctuations which allow to compute the heat capacity curves by using the equation 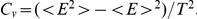 The equilibrium (or melting) temperature 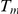 for the folding process corresponds to the peak of the heat capacity curve. The values of 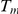 (in reduced units) for all the studied CPs are very close to one another (within a 4% variation).

Since the circular permutation procedure may induce changes in the folding mechanism (see, e.g., [29]), we have also computed the folding free energy profiles at 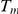 by using the WHAM method [30]. It is worthwhile to mention that we have employed the energy of the system (which, in the lattice G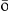 model, is directly related to the fraction of native contacts) as the reaction coordinate to compute the free energy profiles. Despite not being perfect, this choice has proven to be adequate for simple native-centric lattice models as the one considered in this work [31], as well as in off-lattice simulations based on more sophisticated models [32].

**Figure 3:**
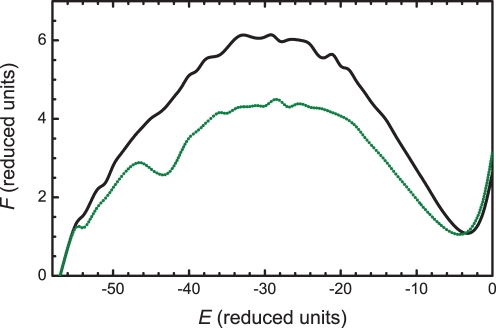
Examples of the free energy profiles *F* obtained for two of the circular permutants in family H. See text for details.

For the majority of CPs belonging to family H and for all the CPs from family L, we get a free energy profile typical of a two-state transition, as illustrated by the solid curve in Figure 3. It shows a narrow minimum located at an energy equal to -57 (in reduced units, corresponding to the 57 native contacts formed), and a wider minimum at energies close to zero (corresponding to the denatured state). For 12 members of family H, however, we have obtained a free energy profile similar to the dotted curve shown in Figure 3. This type of profile indicates the existence of a post-TS intermediate (located at an energy close to -43) in these cases. Although the barriers characterizing the intermediates are small, we have excluded these 12 CPs from the subsequent analysis to keep our study framed to strict two-state folders.

The free energy profiles also allow one to obtain (for different selected temperatures) the free energy difference between the folded and denatured states (i.e., the free energy of folding), and also the free energy difference between the denatured state and the transition state ensemble, TSE (i.e., the activation energy of folding). It is important to mention that this type of analysis provides numerically accurate free energy barriers between the denatured state and the TSE especially at 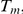 where this barrier attains its maximum value. At lower temperatures, favoring the population of the folded state, the free energy of the unfolded state increases with respect to that of the folded state. This creates a smaller barrier between the unfolded and the transition states, which is more difficult to measure accurately (see, e.g., Fig. 2 in [28]). The free energy barrier between the denatured state and the TSE plays a crucial role in any joint discussion of thermodynamic and kinetic properties. Therefore, all the results presented in this work are computed at the folding equilibrium temperature 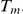


This choice of temperature (which is evaluated independently for each considered CP) creates a difference between the simulation and experimental approaches. Indeed, in experiments *in vitro* [1] the folding rates of different proteins are usually measured at (constant) room temperature. However, and as previously mentioned, the values of 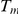 show very little variation across the different CPs investigated in this work. Thus, there is no need to consider a possible temperature dependence of the simulation results reported in the following sections.

## Results and Discussion

### Transition State Theory

**Figure 4:**
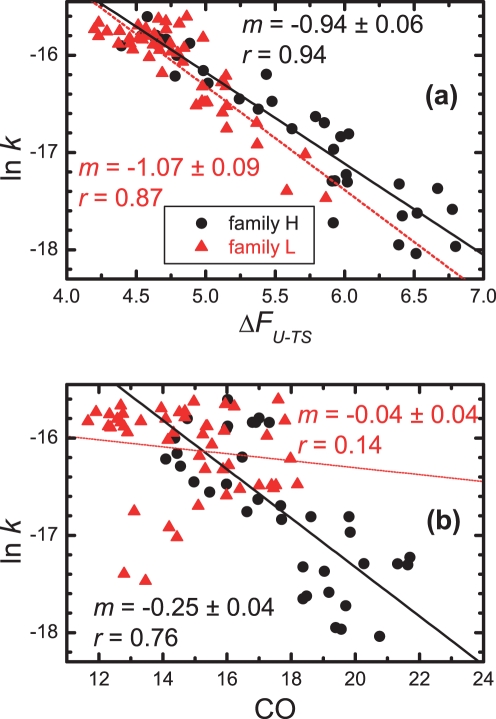
Dependence of the logarithm of the folding rate *k* on (a) the activation energy of folding, and (b) the contact order of the corresponding native structure, for the two families of circular permutants considered in this work. We also report the slopes (*m*) and the absolute values of the correlation coefficients (*r*) for the corresponding linear fits.

A first important result in our combined thermodynamic/kinetic analysis is the existing correlation between the logarithmic folding rate and the activation energy of folding, i.e. the free energy difference between the denatured state and the transition state, 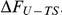 This is reported in Figure 4(a). As it can be observed, the plot reveals very high correlation coefficients and slopes close to -1 in both families H and L. This result should be stressed for two major reasons. Firstly, in general, it is not straightforward to gather accurate kinetic and thermodynamic data in computer simulation studies of protein folding. This limitation results from the exceedingly larger amount of computer time that is required by the use of detailed protein models. The modeling framework adopted here rendered these calculations possible in a rather affordable computational time. Secondly, and more importantly, the results in Figure 4(a) also validate our simulation approach, in particular the use of MC to compute folding rates. If the MC folding time would be depleted from any real dynamic meaning, the protein folding times, which allow the calculation of the corresponding folding rate constants (as shown in Figure 2), would be meaningless. The high correlations shown in Figure 4(a) provide, therefore, a validation of the methodology adopted here.

### Correlation between Lattice Folding Rates and Contact Order

We are now ready to analyze the correlation between the folding rates and the contact order. For our model, these results are shown in Figure 4(b). As one can observe, while the correlation between ln *k* and CO in family H is quite strong, and similar to that reported for real-world two-state proteins (Table 1 of [33]), there is no effective correlation between these two properties in family L. Thus, we could say that in family L the CO does not reflect the activation energy of folding. In order to understand why, and given the high correlations shown in Figure 4(a), which validate the application of transition state theory, one must analyze the transition state ensemble (TSE).

### The Transition State Ensemble

**Figure 5:**
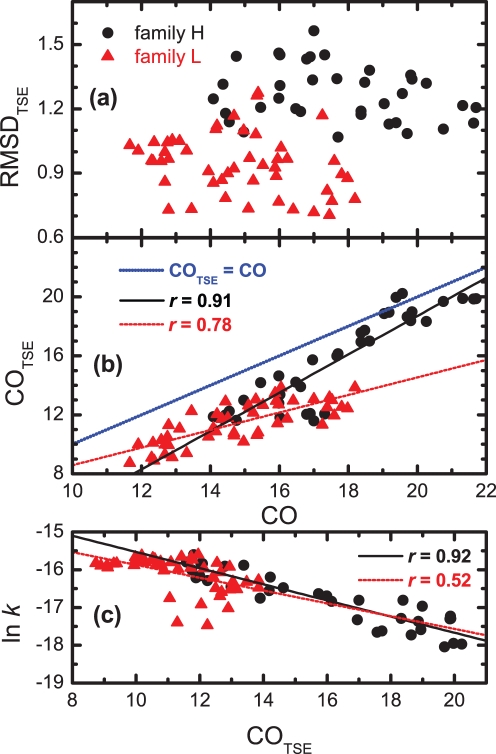
Structural variability in the TSE measured by (a) the RMSD to the native structure (in lattice units) and (b) the relation between the contact order of the TSE and the native contact order. In both families, CO_TSE_ is typically smaller than the CO, a trend that is also observed in real-world proteins [42]. In family H there is a strong correlation between the CO_TSE_ and the CO, despite the considerably large structural variability. In family L, on the other hand, the CO_TSE_ is significantly less correlated with that of the native structure, and in this case the RMSD_TSE_ is significantly lower. (c) Dependence of ln *k* on the average contact order of the transition state ensemble.

For each CP considered in this study we have prepared an ensemble of (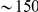) conformations, representative of the TSE, by combining kinetic and thermodynamic information. In particular, the selected conformations have folding probability 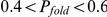 (with error smaller than 5%) [34], [35], and a fraction of native contacts that corresponds to the peak of the free energy profile (i.e., whose value of *F* is between 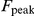 and 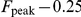); consider the black solid curve in Figure 3 for guidance. Therefore, for every CP we can evaluate two different properties of its TSE: the average CO of the TSE, CO_TSE_, and the average root-mean-square deviation between the TSE structures and the native structure, RMSD_TSE_. This latter property, reported in Figure 5(a), indicates that in the ensembles of structures representing the TSE of each family member, amino acids are on average closer to their native positions in the case of family L than in that of family H. Indeed, the values of the RMSD are systematically larger in the TSEs of family H. However, the fraction of native structure formed in the TSE of the CPs in family L does not statistically reflect the CO of the native conformation (Figure 5(b)). The value for the correlation coefficient *r* between CO_TSE_ and CO for this family is smaller than that observed for family H and the corresponding regression line clearly departs from the blue dotted line (i.e. the identity line) plotted in Figure 5(b). In family H, on the other hand, the CO of the native state is better captured by the TSE, despite a lower overall similarity with the native structure, with values of CO_TSE_ which, at least for half of the members of the family, are very similar to the native COs. The apparently counterintuitive observation that native topology can be achieved in the TSE despite high structural variability was also observed in real-world proteins and rationalized in [36], in the framework of the extended nucleation-condensation mechanism [8], [9]. In this view, the formation of a few contacts in the TSE defines the overall native topology but the zipping of the remaining native contacts that pulls the amino acids into their native positions occurs during the last stage of the folding reaction. Finally, in Figure 5(c) we show the correlation between the logarithmic folding rate (ln *k*) and the average contact order of the TSE. For both families, the ln *k* – CO_TSE_ correlation is substantially stronger than the ln *k* – CO correlation (shown in Figure 4). As mentioned in the Introduction of this manuscript, this observation should not be taken as surprising since the TSE corresponds to the rate-limiting step in folding. However, it should be noted that in the case of family L the ln *k* – CO_TSE_ correlation is still significantly lower. Thus, so far, our analysis shows that the CO correlates well with ln *k* when the CO and the average CO of the TSE are closely related, as it happens in the CPs of family H.

### The Transition State Network

**Figure 6:**
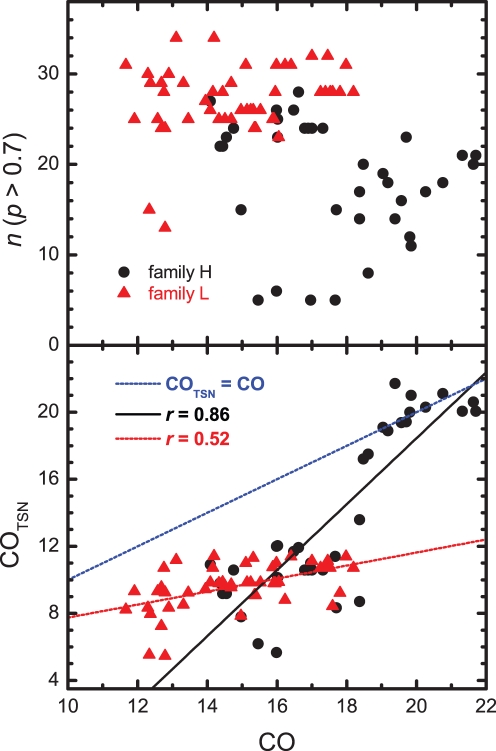
The number of native contacts forming the TSN as a function of CO (top) and the dependence of the contact order of the TSN on the CO (bottom) for families L and H.

In order to understand when and why the CO is captured by the average CO of the TSE, we have restricted the analysis of the relation between CO_TSE_ and CO to the set of native contacts *n* that are present in the TSE with probability 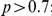 we shall call it *transition state network*, TSN. The number of contacts forming this network is shown in Figure 6(a) as a function of the native contact order. This network should not be confused with the folding nucleus (FN). The TSN represents a larger set of native contacts, which includes the FN, and characterizes the portion or fraction of native structure that is significantly present in the TSE. In general, as it can be seen in Figure 6(a), the size of the TSN is distinctively larger in family L than in family H. We have also computed the average contact order corresponding to the set of contacts forming the TSN. This quantity, which we have named CO_TSN_, is plotted as a function of the native CO in Figure 6(b). In family L the TSNs are characterized by having a large number predominantly local native contacts, as indicated by the relative low values of their CO_TSN_. This observation suggests that the stabilization of the TSE in the case of family L is overwhelmingly energetic, an observation that can explain the modest ln *k* – CO_TSE_ correlation observed in this family. By contrast, in family H, the TSNs are much more heterogeneous, with a wider distribution of CO_TSN_ and *n* for the different CPs in this family (see Figure 6). By identifying exactly which contacts form the TSNs, we have checked that the TSEs in family H are formed by conformations that share a relatively small number of native contacts, of variable range, which is indicative of tertiary structure formation playing a differentiated role in the stabilization of the TSEs of this family. The CO_TSE_ of family H measures the average range of these key native contacts, providing a good metric for the TSE characteristic topology along the family.

Figure 6(b) also shows that the CO is a degenerate parameter with regard to CO_TSN_. This is especially evident in the case of family L. Indeed, in the lower CO range, each CO value may be associated with very different values of CO_TSN_ (e.g. in family L for CO = 12.7, 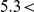
CO
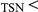
10.6, and in family H for CO = 16, 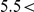
CO
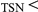
12). This observation is supported by experimental findings reported in [37], where it was shown that two proteins with similar native topology and low CO (apoflavodoxin and CheY) fold via topologically different TSEs. The CO – CO_TSN_ dependence changes sharply when CO >18.5, as seen in Figure 6(b). Indeed, in this high-CO regime, CO_TSN_ takes values close to CO. A close inspection of the corresponding TSNs shows that they are formed by a set of distinctively long-ranged tertiary interactions (with up to 5 native contacts of range 27 and 7 native contacts of range 31 to 49) that do not form in the TSN of the other proteins studied here. This type of long-ranged TSN, whose establishment ensures that the TSE acquires the native topology (note that *r* for the CO – CO_TSE_ dependence drops from 0.91 to 0.73 when the high-CO proteins are removed), is the distinctive feature of family H that makes the folding rate strongly dependent on the CO. Indeed, removing from the analysis all the proteins in family H with CO >18.5 leads to a decrease of the correlation coefficient in the dependence between ln *k* and CO in Figure 4 from 0.75 to 0.48.

### Summary and Final Conclusions

In this work we have used a simple lattice model to analyze the thermodynamic and kinetic characteristics of the folding process for two different families of native structures. The members of each family are related to one another through circular permutation. Our study, in which the effect of the native contact order, CO, has been isolated from other protein properties that are known to affect folding rates, shows that the rate–CO correlation stems from a strong correlation between the CO and the CO of the TSE. It also shows that folding through a TSE exhibiting the native topology occurs when the latter is dominated by a network of interactions which are distinctively long-ranged. This type of TSE occurs mainly in high-CO proteins, because these proteins have predominantly long-range interactions in their native structure, implying the formation of tertiary structure in the TSE. The particular type of TSE identified here, stabilized by a network of long-ranged native interactions and exhibiting a large structural variability, is not specific of lattice proteins. Previous studies based on Molecular Dynamics simulations that used experimental 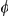-values as restraints to sample TSE conformations showed that two-state proteins src-SH3, spc-SH3, fyn-SH3, AcP, and TNfn3 [38]–[41] fold via a structurally heterogeneous TSE that is also stabilized by an interaction network dominated by similarly long-ranged interactions. Interestingly, these beta-proteins are amongst the two-state proteins with highest CO (%), just like their lattice counterparts identified here [33]. Furthermore, a considerable decrease in the correlation coefficient (11%) is also observed for the ln *k* – CO dependence when these 5 proteins are removed form the dataset (Table 1 of [33]).

Finally, we have found that the correlation between folding rate and the contact order of the TSE may itself be modest in sets of proteins whose TSEs involve predominantly local contacts. In this case the stabilization of the TS is mostly energetic.
